# Oncogenic effect of PHLDB2 is associated with epithelial–mesenchymal transition and E-cadherin regulation in colorectal cancer

**DOI:** 10.1186/s12935-019-0903-1

**Published:** 2019-07-16

**Authors:** Geng Chen, Tong Zhou, Tantan Ma, Tingting Cao, Zhenxiang Yu

**Affiliations:** 1 Department of Gastroenterology, The First Hospital of Jilin University, Changchun, 130021 China; 2 Department of Endocrinology, The First Hospital of Jilin University, Changchun, 130021 China; 3 Department of Respiration, The First Hospital of Jilin University, Xinmin Street, 71, Changchun, 130021 Jilin People’s Republic of China

**Keywords:** PHLDB2, Colorectal cancer, EMT, E-cadherin, Metastasis

## Abstract

**Background:**

Pleckstrin Homology Like Domain Family Member 2 (PHLDB2) is an important protein with a PH-domain for interaction with partners to regulate cell migration. However, the role of PHLDB2 in human cancer metastasis, especially in colon cancer, still remains elusive.

**Methods:**

The RNA-seq and clinical data of colorectal cancer patients from the Cancer Genome Atlas (TCGA) were analyzed for correlations between PHLDB2 and clinical outcomes as well as epithelial–mesenchymal transition (EMT) markers. Wound healing and transwell invasion assays were used to determine the effects of PHLDB2 on cell migration and invasiveness. Western blot and qRT-PCR analyses were employed to detect protein and mRNA changes, respectively. Co-immunoprecipitation was performed to assess protein–protein interaction.

**Results:**

In the present report, by following our previous study, we found that PHLDB2 expression is associated with poorer prognosis, including disease-free survival, tumor stage, nodes pathology, as well as lymphatic and vascular invasion through TCGA data analysis. In addition, PHLDB2 expression is highly correlated with multiple epithelial–mesenchymal transition (EMT) markers involving cell-surface proteins (N-cadherin and OB-cadherin), cytoskeletal markers (α-SMA and Vimentin), ECM proteins (Fibronectin and Laminin 5), and transcription factors (Snail2, ZEB1, and Ets-1). We also demonstrated that PHLDB2 knockdown mediated by siRNA was sufficient to attenuate colon cancer cell migration and invasion, as well as E-Cadherin reduction, by TGF-β treatment. Interestingly, PHLDB2 expression levels were significantly elevated in response to EMT induction by TGF-β and EGF. Moreover, we found that PHLDB2 could bind to MDM2 and facilitate MDM2-mediated E-Cadherin degradation.

**Conclusions:**

Our findings suggest that PHLDB2 is a downstream effector of EMT pathway and may present as an important biomarker for colon cancer prognosis and a target for colon cancer intervention.

**Electronic supplementary material:**

The online version of this article (10.1186/s12935-019-0903-1) contains supplementary material, which is available to authorized users.

## Background

PHLDB2 is a PH domain-containing protein that plays an important role in mediating cell migration by forming complex with its partners, such as CLASPS, Prickle 1 and Liprin α1 [1–3]. In association with its client proteins, PHLDB2 is required for focal adhesion disassembly and cell polarization and migration [1–3]. These functional characteristics suggest a potentially important role of PHLDB2 in cancer cell mobility, and ultimately metastatic progression. However, it remains largely unknown whether and how PHLDB2 is implicated in clinical cancer patients and the underlying mechanisms, especially in colorectal tumors.

Metastasis is the advanced stage of tumor progression that is responsible for cancer mortality in most of cases. Recent studies have demonstrated a critical role of epithelial–mesenchymal transition (EMT) in conferring metastatic traits on cancer cells by facilitating the ability of migrating, invading, and resisting therapies [4, 5]. The EMT program is activated in response to a variety of oncogenic signaling pathways, such as transforming growth factor (TGF), Wnt/β-catenin, and Notch pathways [4, 5]. Better understanding of EMT associated molecular events would provide promising opportunities to develop strategies for improving cancer prognosis and therapy.

In the present study, by analyzing human colorectal cancer database from the Cancer Genome Atlas, we demonstrate not only the association of PHLDB2 expression with patient prognosis, but also the high correlation between PHLDB2 and multiple EMT markers. In addition, we show that PHLDB2 is responsive to EMT induction and at least partially required for cancer cell invasion and migration promoted by TGF-β, likely through interacting with MDM2 to facilitate E-Cadherin degradation. Our findings underscore the importance of PHLDB2 in EMT and metastasis, and therefore provide the rationale for determining PHLDB2 as a biomarker and a target in colorectal cancer.

## Materials and methods

### TCGA data

The colon cancer data were downloaded from cBioportal (TCGA, Provisional) [7, 8]. Analyses were performed on the patients/samples with available data corresponding to each aspect. The Log-rank test was used to determine the disease-free survival. The Pearson correlation coefficient was calculated between PHLDB2 and the EMT markers.

### Cell lines and reagents

Human colon cancer cell line SW620 was purchased from the American Type Culture Collection (ATCC), and HCT116 p53^−/−^ cells were generous gifts from Dr. Bert Vogelstein at the John Hopkins Medical Institutes. The cells were maintained in Dulbecco’s Modified Eagle Medium (DMEM) supplemented with 10% fetal bovine serum, 100 U/ml penicillin and 100 μg/ml streptomycin (Invitrogen), at 37 °C, 5% CO_2_. Human recombinant TGF-β1 was purchased from R&D Systems (Minneapolis, MN). The antibodies were purchased from Santa Cruz (anti-E-cadherin, and anti-Vimentin), Sigma-Aldrich (anti-PHLDB2), Thermo Fisher (anti-MDM2) and Millipore (anti-glyceraldehyde-3-phosphate dehydrogenase, GAPDH). PHLDB2 siRNAs and non-specific control oligo were purchased from Ambion. Transfection was performed using Lipofectamine 2000 (Invitrogen) per manufacture’s instruction.

### Cell invasion and migration assays

Cells were transfected with non-specific control or PHLDB2 siRNA and 24 h after transfection, the cells were replated into Matrigel-coated upper champers (24-well transwell inserts, BD Bioscience) for invasion assay, or into 6-well plates for wound healing migration assay as described previously [6]. Briefly, for the invasion assay, the upper champers were refreshed with serum free medium while the lower champers filled with culture medium containing reconstitution buffer or 5 ng/ml TGF-β at 6 h after replating. The cells were allowed to invade for 48 h, and non-invasive cells were removed with cotton swab and invasive cells were fixed with methanol and stained with crystal violet. Cell numbers were quantitated from 3 different wells. Wound healing assay was performed by comparing the gaps generated with P200 tip scratching between 0 h and 24 h time points.

### Statistical analysis

The student’s two-tailed *t* test was employed to determine the difference between control and treatment groups of cell-based assays (three independent experiments performed for analysis) and different clinical groups. The p value less than 0.05 was considered statistically significant. Data are presented as mean ± SD.

## Results

### PHLDB2 is associated with poorer prognosis of colon cancer patients

**Fig. 1:**
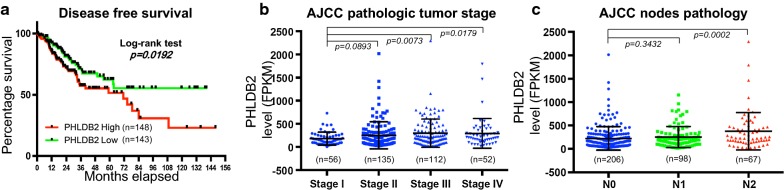
PHLDB2 is associated with poorer clinical outcomes. **a** Disease-free survival. The median expression level of PHLDB2 was used as the cut-off value to define PHLDB2 High or PHLDB2 Low. The Log-rank test was employed to analyze the survival curves. The expression levels of PHLDB2 were presented in different tumor stages (**b**) and different nodes pathology (**c**). p < 0.05 was considered statistically significant

**Fig. 2:**
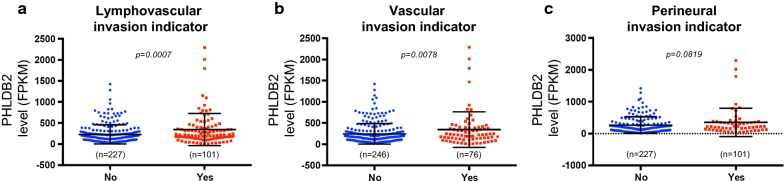
PHLDB2 is associated with colon tumor invasiveness. The expression levels of PHLDB2 were presented in patients with invasion indicators detected (Yes) or not (No) for lymphovascular invasion (**a**), vascular invasion (**b**), or perineural invasion (**c**). p < 0.05 was considered statistically significant

Studies suggest that PHLDB2 is a potential oncogene [1–3, 6], but its clinical implication remains less understood. In order to address this question, we analyzed a set of colorectal adenocarcinoma data deposited by the Cancer Genome Atlas (TCGA) in the cBioportal website [7, 8]. This set included the largest number of patients with RNA-seq data available from TCGA. Consistent with our previous report [6], the disease-free survival of the patients with low PHLDB2 expression (median survival undefined) is significantly longer from those with high PHLDB2 expression (median survival 71.48 months), as shown in Fig. 1a. In line with this result, the expression level of PHLDB2 is associated with pathologic tumor stage (Fig. 1b) and regional lymph nodes pathology (Fig. 1c) defined by American Joint Committee on Cancer (AJCC), as the PHLDB2 levels in Stage III and Stage IV or in N2 are significantly higher than in Stage I or N0, respectively. In the attempt to find association between PHLDB2 and tumor invasiveness, we found that patients with either lymphovascular invasion or vascular invasion also have significantly higher expression of PHLDB2 than those without these indicators (Fig. 2a, b). Although the same analysis does not show significant difference in perineural invasion, there is still a trend of elevated expression of PHLDB2 in perineural invasion indicator positive patients (Fig. 2c). Together, these results indicate that high level of PHLDB2 is associated with poorer clinical outcomes and tumor invasiveness in colorectal cancer patients.

### PHLDB2 expression is correlated with EMT markers

**Fig. 3:**
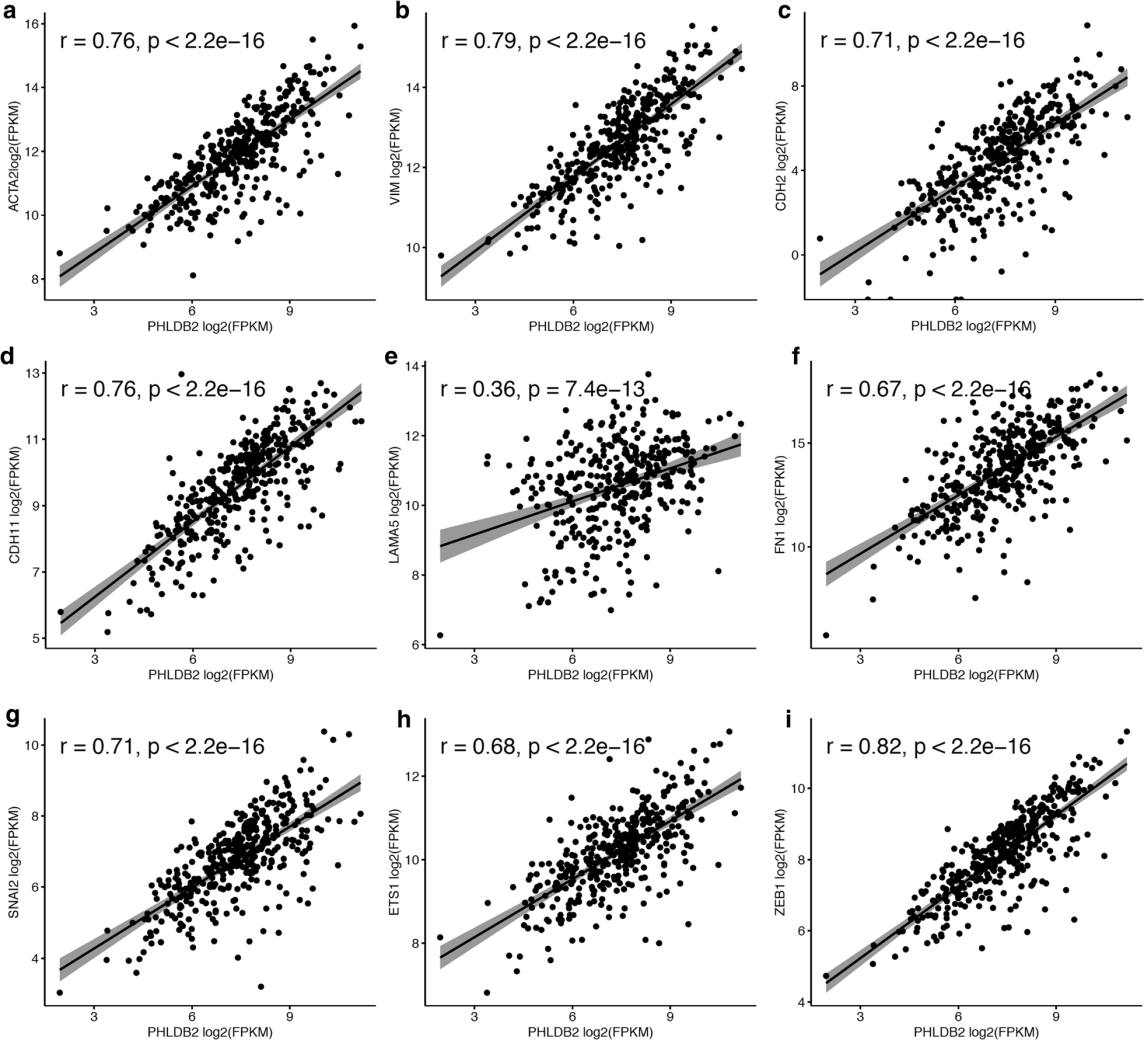
PHLDB2 is positively correlated with multiple EMT markers. The mRNA-seq data were retrieved from TCGA and the Pearson correlation analysis was employed to determine the correlation between PHLDB2 and these EMT markers. r: Pearson correlation coefficient

The epithelial–mesenchymal transition (EMT) is a program activated in certain population of cancer cells to confer metastasis by facilitating cell migration and invasion [4, 5]. While PHLDB2 is an important protein involved in focal adhesion disassembly in migrating cells, we next sought to investigate if PHLDB2 is correlated with any EMT markers in the same set of colorectal cancer patients. Surprisingly, among the well-known EMT markers [5], at least nine of them are highly correlated with PHLDB2 expression. As shown in Fig. 3, the Pearson correlation analysis demonstrates that these EMT markers include cytoskeletal markers [α-SMA (ACTA2) and Vimentin (VIM)], cell surface proteins [N-cadherin (CDH2) and OB-Cadherin (CDH11)], extracellular matrix (ECM) proteins [Laminin 5 (LAMA5) and Fibronectin (FN1)], and transcription factors [Snail2 (SNAI2), ZEB1, and Ets-1 (ETS1)]. The correlation was especially high with cytoskeletal markers (Fig. 3a, b), cell surface proteins (Fig. 3c, d), and transcription factors (Fig. 3g–i). These findings indicate that PHLDB2 highly likely plays an important role in mediating EMT process.

### PHLDB2 depletion attenuates the EMT promoting effects of TGF-β

**Fig. 4:**
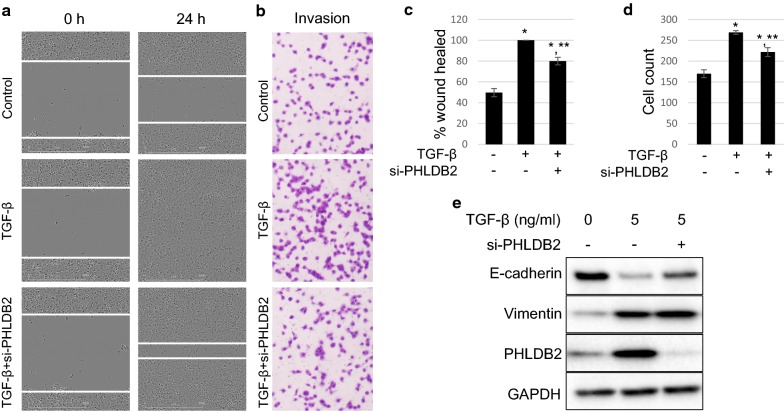
PHLDB2 knockdown attenuates TGF-β induced cell migration, invasion, and E-Cadherin reduction. **a** HCT116 p53^−/−^ cells were transfected with non-specific control siRNA and siRNA against PHLDB2 at 40 nM. 24 h after transfection, the cells were replated into 6-well plates to attach for 6 h and the scratch was made for 0 h time point. Then the cells were treated with vehicle or 5 ng/ml TGF-β for another 24 h for imaging. **b** HCT116 p53^−/−^ cells were transfected with non-specific control siRNA and siRNA against PHLDB2 at 40 nM. 24 h after transfection, the cells were replated into Matrigel-coated upper champers (24-well transwell inserts) and allowed to attach for 6 h before vehicle or 5 ng/ml TGF-β were added to the lower wells for another 24 h and subjected to invasion assay. **c** Quantitation of 3 independent wound healing assays. **d** Quantitation of 3 independent invasion assays. **e** Cells mentioned in (**a**) and (**b**) were harvested in parallel for Western Blot analysis. *p < 0.05 as compared with vehicle control. **p < 0.05 as compared with TGF-β alone

As we have demonstrated the correlation of PHLDB2 with multiple EMT markers in clinical data, we next sought to further validate the role of PHLDB2 in EMT in cell-based assays. TGF-β signaling is an important pathway in inducing EMT phenotype [9]. To test the function of PHLDB2 in TGF-β-induced EMT, we treated HCT116 p53^−/−^ cells with TGF-β while knocking down PHLDB2 using siRNA and subjected the cells to wound healing assay and transwell invasion assay. As shown in Fig. 4a, c, at 24 h after scratching, TGF-β treatment promoted the wound gap to a full closure, while the control cells were only half-way closed. Markedly, PHLDB2 knockdown resulted in ~ 80% heal in the presence of TGF-β, and significantly mitigated TGF-β effect. To confirm that this effect was achieved specifically by PHLDB2 knockdown, we employed another siRNA against the non-coding region of PHLDB2 (si-PHLDB2x) and re-introduced Flag-PHLDB2 into cells. The results shown in Additional file 1: Figure S1 clearly demonstrated that si-PHLDB2x also significantly attenuated TGF-β induced cell migration, which was rescued by re-introducing exogenous PHLDB2. Similar results were observed in another colon cancer cell line SW620 (Additional file 1: Figure S2). In line with the wound healing assay, TGF-β treatment induced ~ 58% more invasive cells compared to control group, while this induction was significantly attenuated by PHLDB2 depletion (Fig. 4b, d). Interestingly, while TGF-β treatment clearly suppressed E-cadherin expression, and induced Vimentin expression, it also caused a significant increase in PHLDB2 protein level (Fig. 4e). Meanwhile, PHLDB2 knockdown resulted in less effective reduction of E-cadherin by TGF-β (Fig. 4e). Collectively, these data suggest that PHLDB2 is indeed an important mediator of EMT, likely in the downstream of TGF-β pathway, as PHLDB2 depletion at least partially compromised TGF-β-induced EMT phenotypes as well as E-cadherin inhibition.

### PHLDB2 expression is responsive to EMT induction

**Fig. 5:**
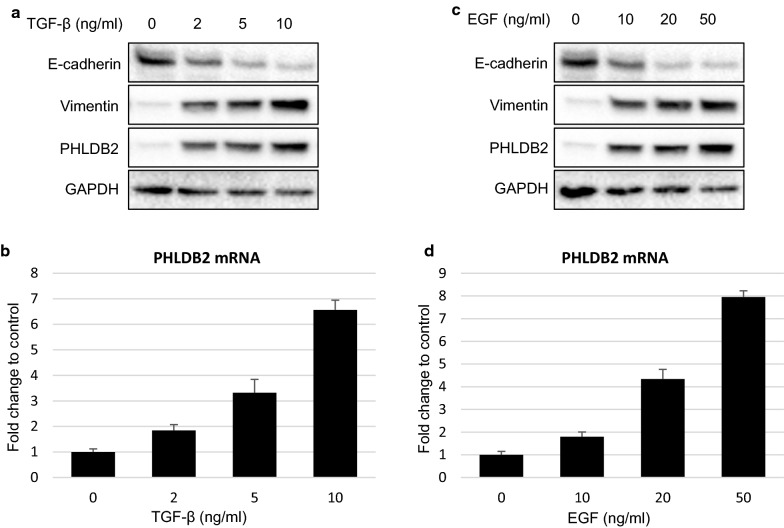
PHLDB2 expression is elevated in response to EMT induction. HCT116 p53^−/−^ cells were treated with TGF-β (**a**, **b**) or EGF (**c, d**) at indicated concentrations for 24 h and subjected to Western Blot (**a**, **c**) and qRT-PCR (**b**, **d**) analysis

The observation from Fig. 4e led us to further evaluate the change of PHLDB2 expression in response to EMT induction. We treated HCT116 p53^−/−^ cells with increasing concentrations of TGF-β or epidermal growth factor (EGF) for 24 h and collected cell lysates for Western Blot and qRT-PCR analysis. As shown in Fig. 5a, in addition to reducing E-cadherin and inducing Vimentin protein expression, TGF-β also up-regulated PHLDB2 protein level in a dose-dependent manner. Up-regulation of PHLDB2 by TGF-β also occurred at mRNA level (Fig. 5b). Similarly, EGF treatment also resulted in a dose-dependent induction of PHLDB2 protein and mRNA expression (Fig. 5c, d). In line with our previous finding, these data suggest that in colon cancer cells, EMT induction by two important pathways, TGF-β and EGF, transcriptionally activates PHLDB2 expression.

### PHLDB2 binds to MDM2 and facilitates MDM2-mediated E-cadherin degradation

**Fig. 6:**
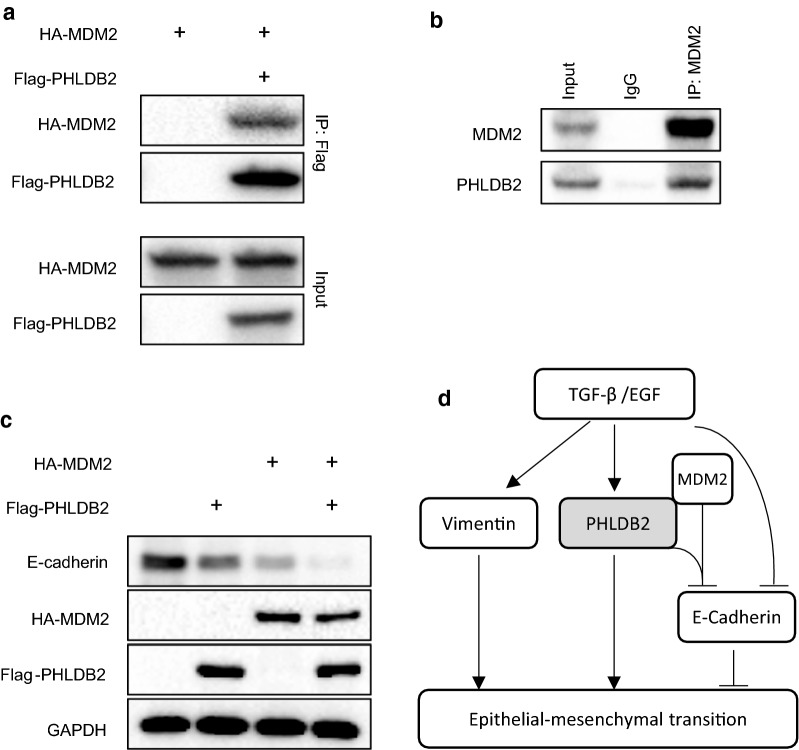
PHLDB2 binds to MDM2 and facilitates MDM2-mediated E-Cadherin degradation. **a** HCT116 p53^−/−^ cells were transfected with HA-MDM2 with or without Flag-PHLDB2 and cell lysis was harvested for co-immunoprecipitation using anti-Flag antibody and Western Blot analysis at 48 h post-transfection. **b** HCT116 p53^−/−^ cells were lysed and subjected to co-immunoprecipitation using anti-MDM2 antibody or mouse IgG as negative control. **c** HCT116 p53^−/−^ cells were co-transfected with plasmids as indicated and subjected to Western Blot analysis at 48 h post-transfection. **d** Scheme depicts the role of PHLDB2 in EMT pathway. EMT stimuli, for example TGF-β or EGF, regulate multiple genes expression, such as Vimentin, E-Cadherin, and PHLDB2 in our study. PHLDB2 can bind to MDM2 to facilitate E-Cadherin degradation, and therefore promote EMT phenotypes

Mouse double minute 2 (MDM2) has been reported to promote EMT by negatively regulating E-cadherin expression through ubiquitination and degradation [10]. Considering our observation in Fig. 4e that PHLDB2 knockdown attenuated the reduction of E-cadherin by TGF-β, we next sought to test if PHLDB2 had any function in regulating E-cadherin expression involving MDM2. We transfected HCT116 p53^−/−^ cells with HA-MDM2 in the absence or the presence of Flag-PHLDB2 and performed co-immunoprecipitation assay using anti-Flag antibody. Interestingly, the result in Fig. 6a showed that these two exogenous proteins indeed bound to each other. We further used anti-MDM2 antibody for endogenous protein and the result confirmed the interaction between PHLDB2 and MDM2 (Fig. 6b). In order to test the effect of PHLDB2 on MDM2-mediated E-cadherin expression, we then transfected HCT116 cells with HA-MDM2 or Flag-PHLDB2 either alone or together, and found that E-cadherin protein was reduced by HA-MDM2 or Flag-PHLDB2 alone and the reduction was more profound when both HA-MDM2 and Flag-PHLDB2 were present (Fig. 6c). Together, these observations indicate that by interacting with MDM2, PHLDB2 could facilitate MDM2-mediated E-cadherin down-regulation.

## Discussion

Epithelial mesenchymal transition (EMT) is a dynamic, transient, and reversible process occurring in a certain population of tumor cells, which is characterized by the loss of epithelial cell traits while the gain of mesenchymal cell properties [4]. Associated with EMT are changes of an array of genes in different but orchestrated functional categories, including transcription factors, microRNAs, cytoskeletal proteins, cell-surface proteins, as well as extracellular matrix proteins [5]. During EMT process, cancer cells lose their polarity and cell adhesion, and acquire the ability to migrate and invade, resulting in tumor metastasis [4, 5]. Given the important role of EMT in metastasis and the present obstacles in discovering effective therapies targeting EMT, it still remains an active area to study EMT associated events.

Previous studies have shown that PHLDB2, as an important component in a complex with other partners, participates in focal adhesion turnover, facilitating cell–matrix interaction to drive tumor cell migration [1–3]. Our previous report also indicates that PHLDB2 overexpression counteracts the inhibitory effects of miR-29c-3p in cell migration and invasion, and also is associated with poorer metastasis-free survival of colon cancer patients [6].

In the present study, we expanded our analysis to the database retrieved from TCGA with the largest number of colorectal cancer patients available. In support of our previous findings, we observed worse outcomes in several clinical categories, including disease-free survival, AJCC tumor stages and nodes pathology, and lymphovascular and vascular invasion, in patients with higher PHLDB2 expression (Figs. 1, 2). More importantly, in an attempt to assess the relationship between PHLDB2 and EMT, we found that PHLDB2 expression levels are highly correlated with multiple EMT markers in almost all the categories mentioned above, e.g., cell-surface proteins (N-cadherin and OB-cadherin), cytoskeletal markers (α-SMA and Vimentin), ECM proteins (Fibronectin and Laminin 5), and transcription factors (Snail2, ZEB1, and Ets-1) (Fig. 3). In addition, we further validated the role of PHLDB2 in EMT and cell mobility by knocking down PHLDB2 in the presence of TGF-β, an inducer of EMT phenotypes. The results clearly showed that PHLDB2 depletion attenuated colon cancer cell migration and invasion facilitated by TGF-β (Fig. 4).

Intriguingly, by using two EMT promoting agents, TGF-β and EGF, we demonstrated that PHLDB2 expression at both mRNA and protein levels is responsive to EMT induction (Fig. 5), indicating PHLDB2 as a downstream target of EMT pathways. Functionally, PHLDB2 binds to MDM2 and facilitates MDM2-mediated E-cadherin down-regulation (Fig. 6a–c). Our findings therefore provide another mechanism by which PHLDB2 acts as an important oncogenic protein to promote EMT and metastatic phenotypes (Fig. 6d).

As the upstream events regulating PHLDB2 are less well studied, it would be very interesting in the future to find out which oncogenic signaling pathway could induce PHLDB2 expression, or any one of the EMT-associated transcription factors could transcriptionally target PHLDB2 since Snail2, ZEB1 and Ets-1 are all highly correlated with PHLDB2 expression.

## Conclusions

Our present study revealed the important role of PHLDB2 in clinical outcomes and EMT from a large cohort of colorectal patients, and therefore provide the rationale to determine PHLDB2 as a valuable biomarker and/or to develop strategies to target PHLDB2 for colorectal cancer intervention.

## Additional file

**Additional file 1: Figure S1.** PHLDB2 overexpression rescues the inhibition of TGF-β induced cell migration by PHLDB2 knockdown. (A) HCT116 p53^−/−^ cells were transfected with non-specific control siRNA and siRNA against PHLDB2 (Non-coding region) at 40 nM.  24 h after transfection, the cells were transfected with vector plasmid or Flag-PHLDB2 and 24 h later the cells were replated into 6-well plates to attach for 6 h and the scratch was made for 0 h time point. Then the cells were treated with vehicle or 5ng/ml TGF-β for another 24 h for imaging. (B) Western blot analysis confirms re-introduction of Flag-PHLDB2. (C) Quantitation of 3 independent scratch assays on HCT116 p53^−/−^ cells. * p<0.05 as compared with vehicle control.  ** p<0.05 as compared with TGF-β alone. ∆: p<0.05, compared with si-PHLDB2x. si-PHLDB2x, siRNA targeting the non-coding region of PHLDB2 mRNA. **Figure S2.** PHLDB2 knockdown attenuates SW620 cell migration induced by TGF-β. (A) SW620 cells were transfected with non-specific control siRNA and 2 different siRNAs against PHLDB2 at 40 nM, and subjected to scratch assay as described in Figure S1A. (B) Quantitation of 3 independent scratch assays. * p<0.05 as compared with vehicle control.  ** p<0.05 as compared with TGF-β alone. si-PHLDB2x, siRNA targeting the non-coding region of PHLDB2 mRNA.

## Data Availability

All data generated or analyzed during this study are included in this published article.

## Abbreviations

PHLDB2: Pleckstrin Homology Like Domain Family Member 2
TCGA: the Cancer Genome Atlas
EMT: Epithelial–mesenchymal transition
TGF: transforming growth factor
EGF: epidermal growth factor
ATCC: American Type Culture Collection
DMEM: Dulbecco’s Modified Eagle Medium
AJCC: American Joint Committee on Cancer
