# Supplementary material for: Oncogenic effect of PHLDB2 is associated with epithelial–mesenchymal transition and E-cadherin regulation in colorectal cancer
Source: Cancer Cell Int. 2019 Jul 16;19:184. doi: 10.1186/s12935-019-0903-1 (PMC6636018; doi:10.1186/s12935-019-0903-1)
Supplement: Supplementary file 1 — Additional file 1: Figure S1. PHLDB2 overexpression rescues the inhibition of TGF-β induced cell migration by PHLDB2 knockdown. (A) HCT116 p53−/− cells were transfected with non-specific control siRNA and siRNA against PHLDB2 (Non-coding region) at 40 nM. 24 h after transfection, the cells were transfected with vector plasmid or Flag-PHLDB2 and 24 h later the cells were replated into 6-well plates to attach for 6 h and the scratch was made for 0 h time point. Then the cells were treated with vehicle or 5ng/ml TGF-β for another 24 h for imaging. (B) Western blot analysis confirms re-introduction of Flag-PHLDB2. (C) Quantitation of 3 independent scratch assays on HCT116 p53−/− cells. * p<0.05 as compared with vehicle control. ** p<0.05 as compared with TGF-β alone. ∆: p<0.05, compared with si-PHLDB2x. si-PHLDB2x, siRNA targeting the non-coding region of PHLDB2 mRNA. Figure S2. PHLDB2 knockdown attenuates SW620 cell migration induced by TGF-β. (A) SW620 cells were transfected with non-specific control siRNA and 2 different siRNAs against PHLDB2 at 40 nM, and subjected to scratch assay as described in Figure S1A. (B) Quantitation of 3 independent scratch assays. * p<0.05 as compared with vehicle control. ** p<0.05 as compared with TGF-β alone. si-PHLDB2x, siRNA targeting the non-coding region of PHLDB2 mRNA. [file 12935_2019_903_MOESM1_ESM.pptx]

## Slide 1
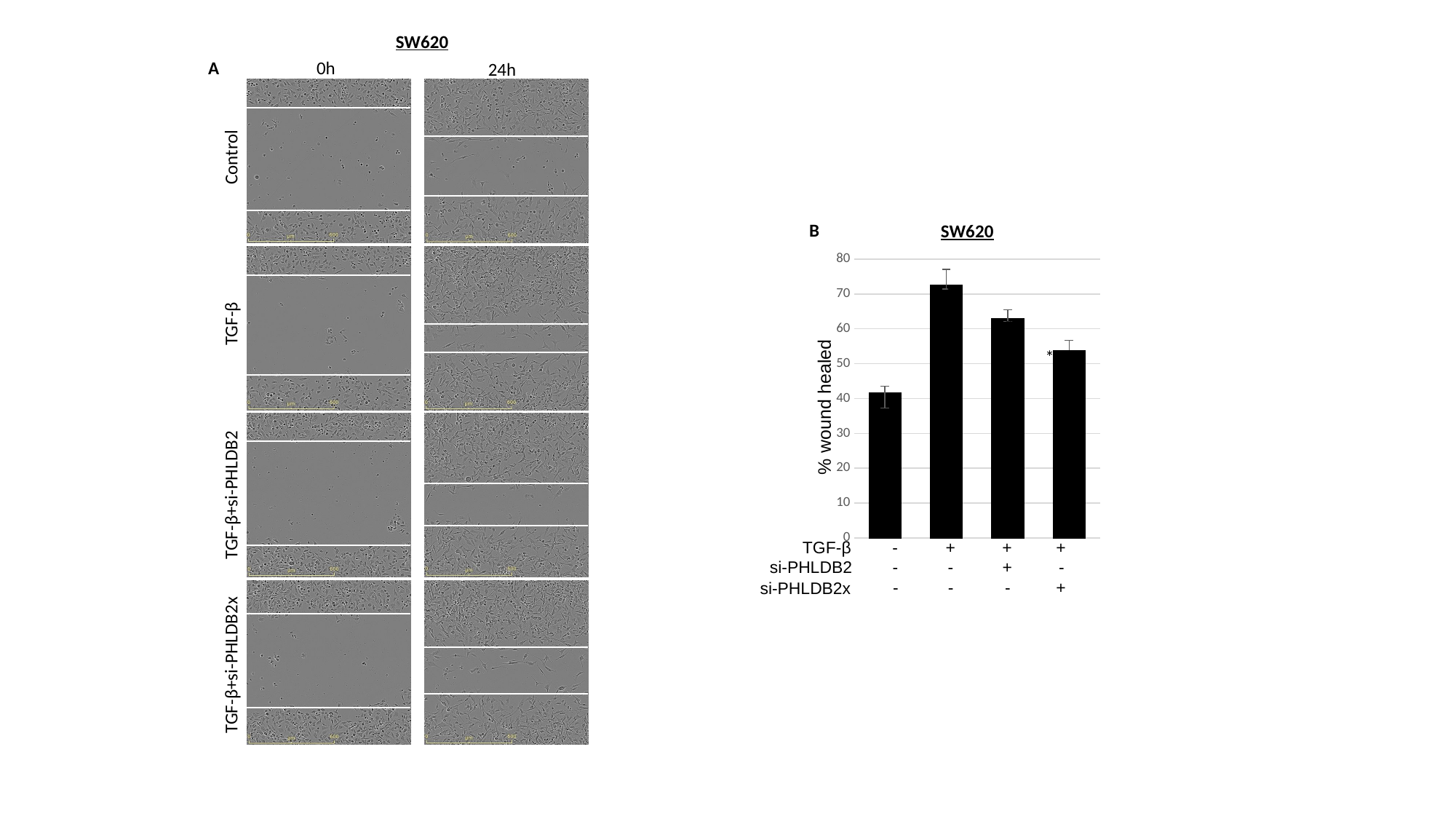

SW620
A
0h
24h
Control
B
SW620
### Chart
| Category | |
|---|---|
| Ctrl | 41.736554369293295 |
| TGF-b | 72.55755516625082 |
| TGF-b+si-PHLDB2 | 63.05857487922706 |
*
TGF-β
*,**
*,**
% wound healed
TGF-β+si-PHLDB2
+
+
+
-
TGF-β
si-PHLDB2
-
-
+
-
-
-
+
-
si-PHLDB2x
TGF-β+si-PHLDB2x

## Slide 2
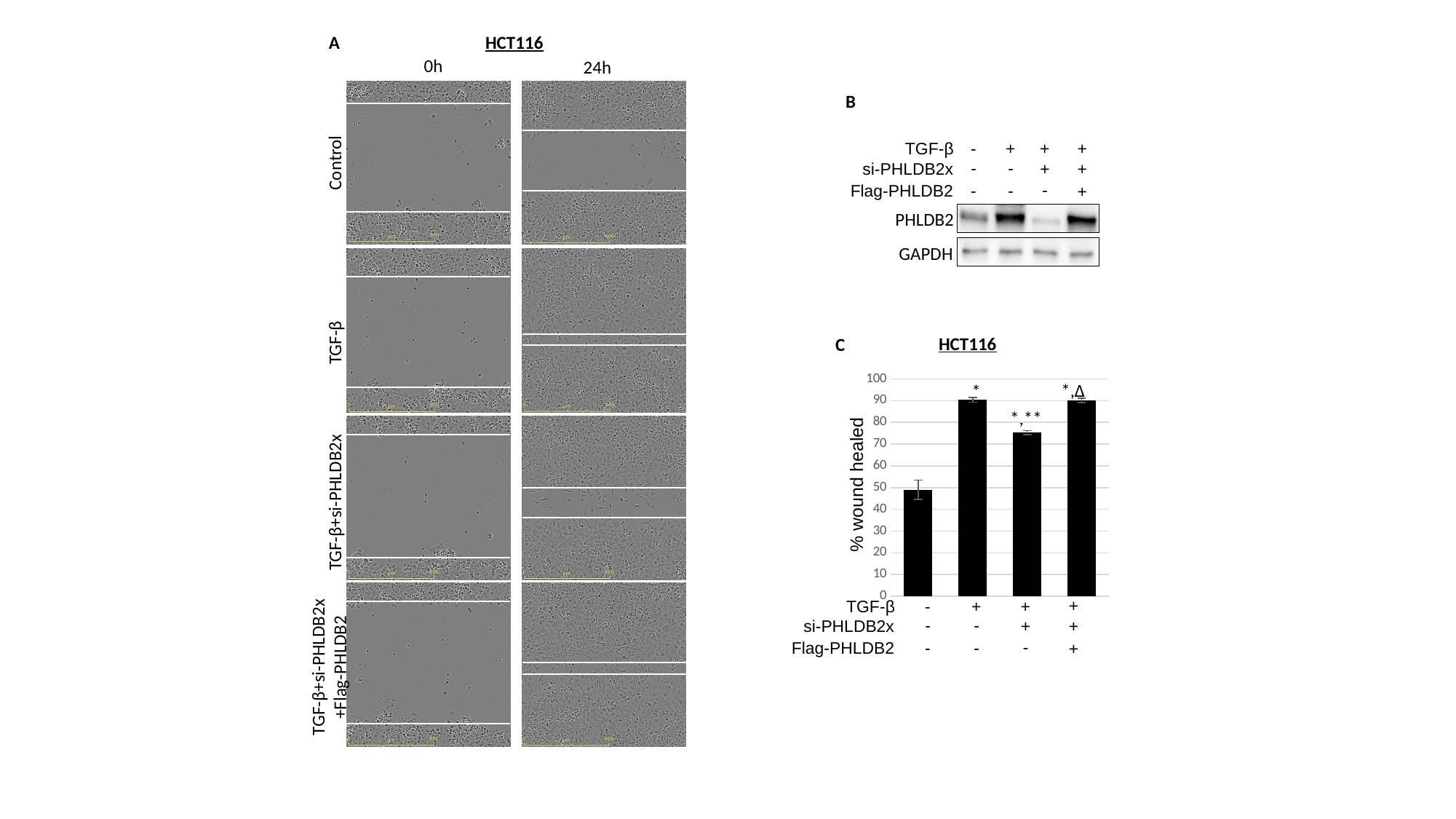

HCT116
A
0h
24h
B
+
+
+
-
TGF-β
Control
-
-
si-PHLDB2x
+
+
-
Flag-PHLDB2
-
-
+
PHLDB2
GAPDH
TGF-β
HCT116
C
### Chart
| Category | |
|---|---|
| Ctrl | 48.932461873638346 |
| TGF-b | 90.36701139749603 |
| TGF-b+si-PHLDB2 | 75.30277245806714 |*,∆
*
*,**
% wound healed
TGF-β+si-PHLDB2x
+
+
+
-
TGF-β
-
-
si-PHLDB2x
+
+
-
Flag-PHLDB2
-
-
+
TGF-β+si-PHLDB2x
+Flag-PHLDB2
